# Supplementary material for: Thermal proteome profiling of breast cancer cells reveals proteasomal activation by CDK4/6 inhibitor palbociclib
Source: EMBO J. 2018 Apr 18;37(10):e98359. doi: 10.15252/embj.201798359 (PMC5978322; doi:10.15252/embj.201798359)
Supplement: Supplementary file 1 — Expanded View Figures PDF [file EMBJ-37-e98359-s001.pdf]

Expanded View Figures

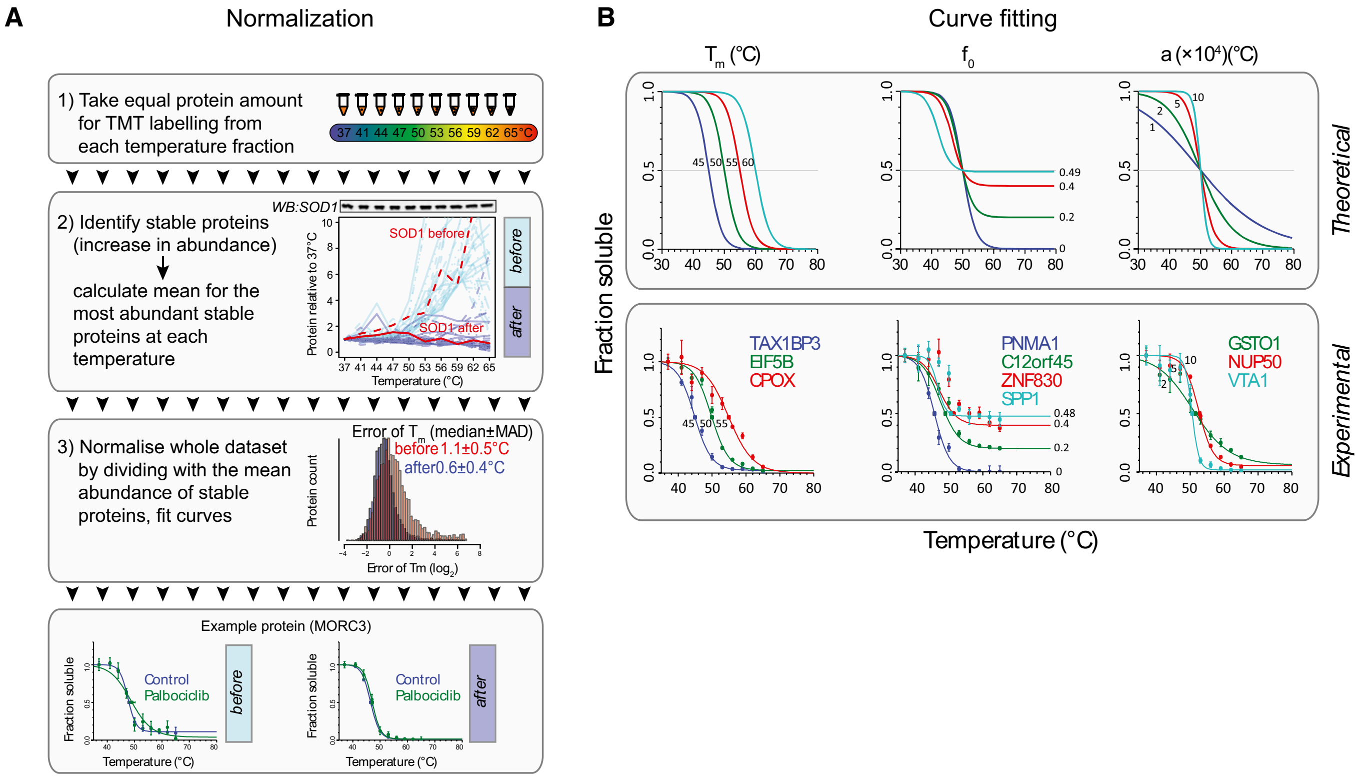

Figure EV1. Data analysis of thermal proteome profiling.

A Data normalization. Equal amounts of total protein were taken from each temperature point for analysis by mass spectrometry. Thermally stable proteins were identified by increased abundance. The known thermally stable protein SOD1 was within the 32 most abundant and stable proteins. Histogram depicts the effect of the normalization procedure for the median T_m error for the whole dataset.

B Curve fitting. Example proteins with different T_m, f_0 and a values are shown below the theoretical curves to show the diversity of denaturation profiles in the MCF7 proteome.

Data information: In individual protein, denaturation profiles the data are presented as means ± SEM from two or three individual biological replicates.

Figure EV2. The denaturation profiles for the human proteins are intrinsic and largely cell type independent.

A Comparison of the T_m values measured for MCF7 breast cancer cells with the previous dataset in K562 leukemic cells. Pearson correlation r = 0.75. A number of outliers are highlighted (see panel C).

B Same as (A), but correlation of MCF7 cell data with analysis from lysed K562 cells. Note the substantially lower correlation.

C Denaturation curve fitting for the outliers shown in (A) in MCF7 cells. Individual data points for K562 cells data are shown in red for comparison. No error is reported for K562 data in the original publication.

Data information: In individual protein denaturation profiles, the data are presented as means ± SEM from two or three individual biological replicates.

▶

© 2018 The Authors

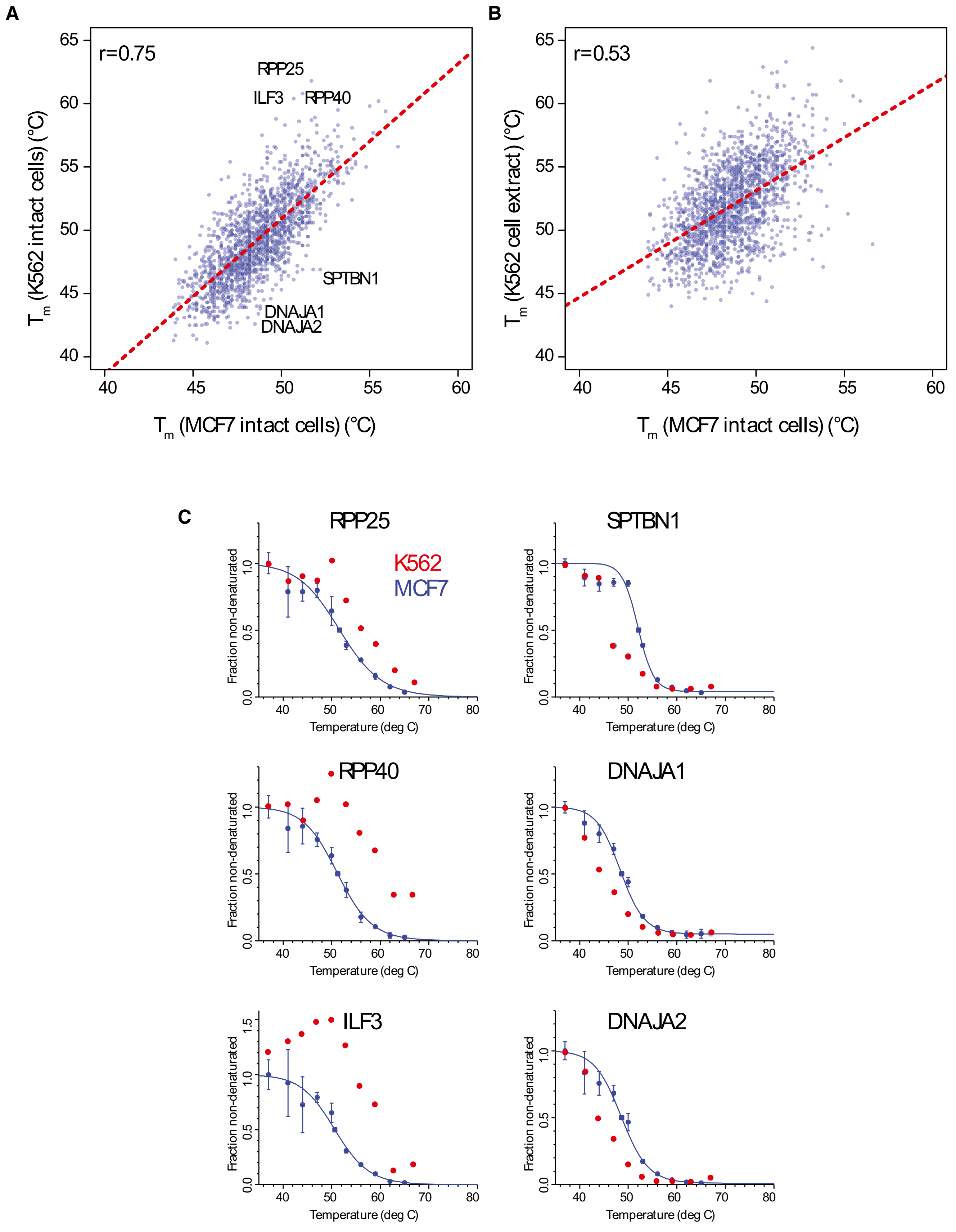
A
r=0.75
RPP25
ILF3
RPP40
SPTBN1
DNAJA1
DNAJA2
T_m (K562 intact cells) (°C)
T_m (MCF7 intact cells) (°C)
B
r=0.53
T_m (K562 cell extract) (°C)
T_m (MCF7 intact cells) (°C)
C
RPP25
K562
MCF7
SPTBN1
RPP40
DNAJA1
ILF3
DNAJA2
Fraction non-denaturated
Temperature (deg C)

Figure EV2.

Figure EV3. Thermal profiling identifies kinases involved in PI3K/AKT/mTOR signaling and glycolytic metabolism as palbociclib targets.

A Palbociclib-induced negative thermal shift in CDK7 displayed by Western blot.

B Thermal denaturation curves of the indicated kinases, all of which displayed clear thermal shifts in the presence of palbociclib (n = 2–3).

C Comparison of top kinases identified based on ΔT_m and *in vitro* kinase activity assay results for the corresponding kinases. The kinases with high ΔT_m and low *in vitro* activity in the presence of palbociclib are likely to be direct targets.

D Schematic of the PI3K/AKT/mTOR signaling pathway and its connection to glycolysis and cyclin D. The components in red displayed a positive ΔT_m and may represent direct targets for palbociclib. The components in orange displayed a negative ΔT_m and are putative indirect targets for palbociclib.

E Changes in phosphorylation levels of mTOR Ser2448 and ribosomal protein S6 Ser235/236 site in T47D cells incubated with indicated concentrations of palbociclib for 1 h. Measurement was performed with the Pathscan Intracellular Signaling antibody array (n = 4).

F Western blots of MCF7 cells displaying the phosphorylation status of components in the PI3K/AKT/mTOR signaling pathway. The cells were first starved for 18 h, then treated with increasing concentrations palbociclib for 5 h or with indicated positive controls for 0.5 h, after which insulin was added for 0.5 h before sample collection.

G Measurements of extracellular acidification rate (glycolysis) and oxygen consumption in MCF7 cells as a function of time. Palbociclib or control was injected to the cells after first four measurements (dashed green line) (n = 5–6). This metabolic phenotype induced by higher concentrations of palbociclib is consistent with the PI3K/AKT/mTOR inhibition.

Data information: In panels (B and G), data are presented as means ± SEM, and in panel (E), data are presented as means ± SD; each n represents an individual biological replicate. In panel (G), data are normalized to control at each time point.

© 2018 The Authors

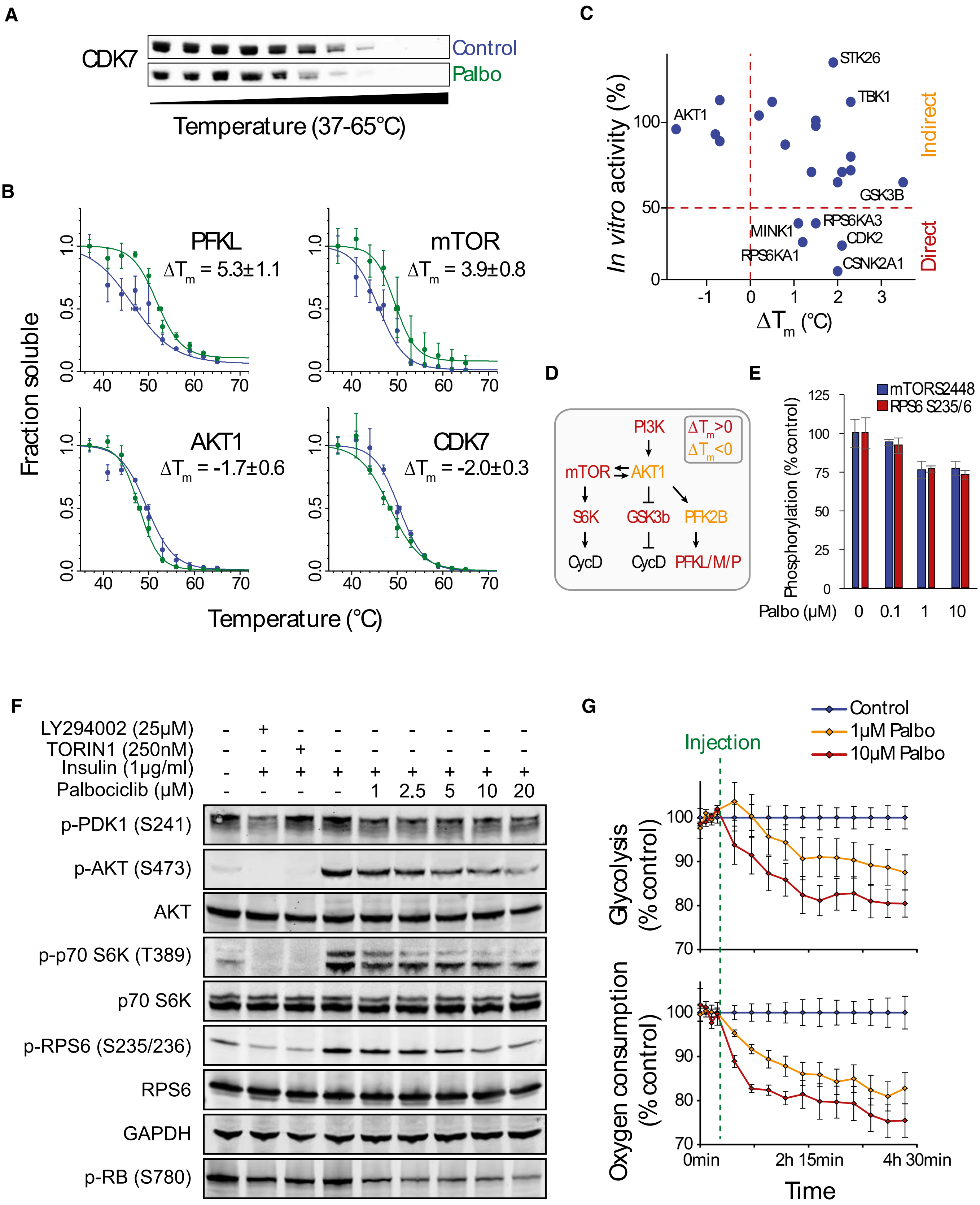
A
CDK7
Control
Palbo
Temperature (37-65°C)
B
Fraction soluble
PFKL
ΔTm = 5.3±1.1
mTOR
ΔTm = 3.9±0.8
AKT1
ΔTm = -1.7±0.6
CDK7
ΔTm = -2.0±0.3
Temperature (°C)
C
In vitro activity (%)
ΔTm (°C)
STK26
TBK1
AKT1
GSK3B
MINK1
RPS6KA3
CDK2
RPS6KA1
CSNK2A1
Indirect
Direct
D
PI3K
ΔTm>0
ΔTm<0
mTOR
AKT1
S6K
GSK3b
PFK2B
CycD
CycD
PFKL/M/P
E
Phosphorylation (% control)
mTORS2448
RPS6 S235/6
Palbo (μM) 0 0.1 1 10
F
LY294002 (25μM)
TORIN1 (250nM)
Insulin (1μg/ml)
Palbociclib (μM)
1 2.5 5 10 20
p-PDK1 (S241)
p-AKT (S473)
AKT
p-p70 S6K (T389)
p70 S6K
p-RPS6 (S235/236)
RPS6
GAPDH
p-RB (S780)
G
Control
1μM Palbo
10μM Palbo
Injection
Glycolysis (% control)
Oxygen consumption (% control)
0min
2h 15min
4h 30min
Time

Figure EV3.

Figure EV4. Palbociclib increases proteasome activity and the clearance of protein aggregates without significant effects on autophagy.

A Proteasome activity in T47D cells, as measured using Me4BodipyFL-Ahx3Leu3VS probe. Cells were treated with the indicated compounds for 1 h followed by 1 h with the probe. 50 nM bortezomib was used as a negative control. Fluorescence was quantified using flow cytometry (n = 3).

B Proteasome activity in HeLa and MCF7 cells after 24-h treatment with 1 μM palbociclib, as measured using Me4BodipyFL-Ahx3Leu3VS probe (n = 3–4).

C Western blots of autophagy markers LC3A/B and SQSTM1/p62 after 6-h treatment with indicated chemicals. Bafilomycin, a v-ATPase inhibitor inhibiting autophagosome fusion with the lysosome, and TORIN-1, an autophagy activator, were used as controls.

D Quantifications of the protein levels in panel (C). Note that TORIN-1 and bafilomycin used as controls inhibit and induce autophagy, respectively.

E Top, Schematic of the experimental setup. The cells were treated for 18 h with 5 μM MG-132 to induce the accumulation of protein aggregates after which cells were washed and 4 h later the remaining amount of protein aggregates was analyzed. Bottom, Quantifications of protein aggregate levels (n = 4).

F Proteasome activity in each cell cycle phase in the presence and absence of palbociclib as measured by the Me4BodipyFL-Ahx3Leu3VS probe and DNA staining (propidium iodide) in HeLa cells (n = 3).

Data information: In panels (A, B, E, F), data are presented as means ± SD; each n represents an individual biological replicate.

▶

 © 2018 The Authors

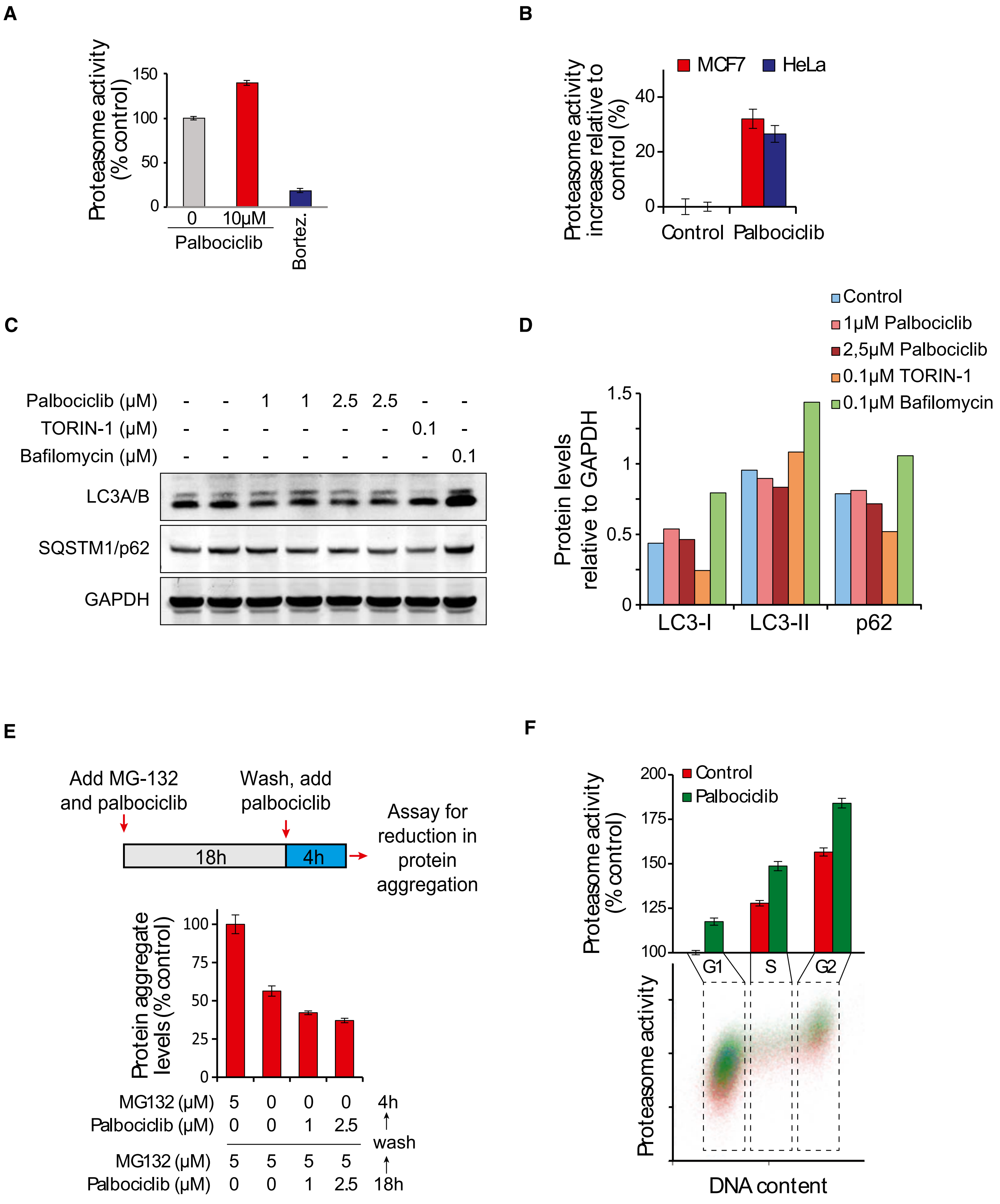
A
Proteasome activity (% control)
150
100
50
0
0
10μM
Palbociclib
Bortez.
B
MCF7
HeLa
Proteasome activity increase relative to control (%)
40
20
0
Control
Palbociclib
C
Palbociclib (μM) - - 1 1 2.5 2.5 - -
TORIN-1 (μM) - - - - - - 0.1 -
Bafilomycin (μM) - - - - - - - 0.1
LC3A/B
SQSTM1/p62
GAPDH
D
Control
1μM Palbociclib
2,5μM Palbociclib
0.1μM TORIN-1
0.1μM Bafilomycin
Protein levels relative to GAPDH
1.5
1
0.5
0
LC3-I
LC3-II
p62
E
Add MG-132 and palbociclib
Wash, add palbociclib
18h
4h
Assay for reduction in protein aggregation
Protein aggregate levels (% control)
100
75
50
25
0
MG132 (μM) 5 0 0 0 4h
Palbociclib (μM) 0 0 1 2.5
wash
MG132 (μM) 5 5 5 5
Palbociclib (μM) 0 0 1 2.5 18h
F
Control
Palbociclib
Proteasome activity (% control)
200
175
150
125
100
G1
S
G2
Proteasome activity
DNA content

Figure EV4.

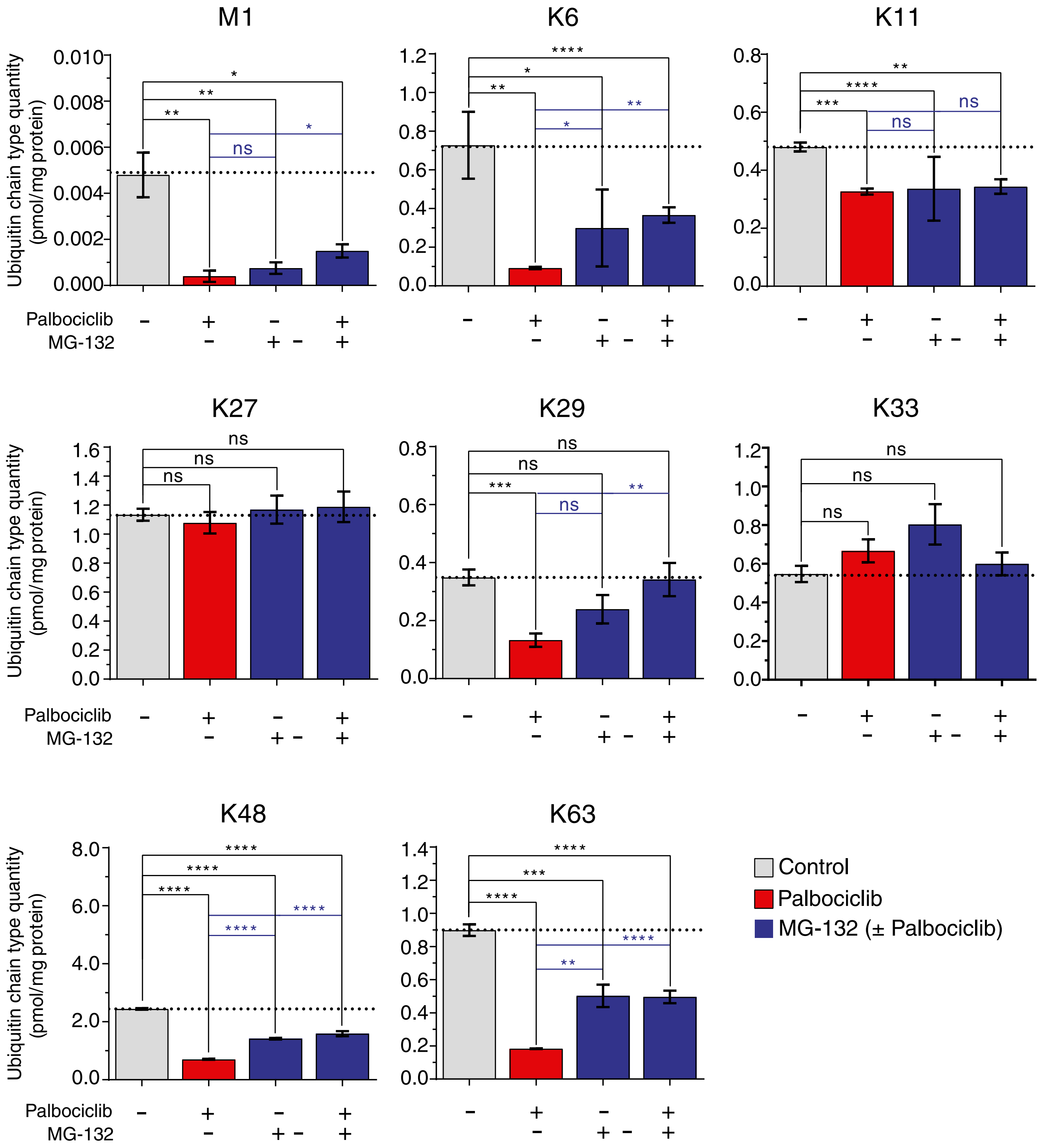

Figure EV5. Targeted mass spectrometry of enriched polyubiquitin linkage types (M1, K6, K11, K27, K29, K33, K48, and K63) after 1-h treatment of MCF7 cells with 1 μM palbociclib, and/or 10 μM MG- 132.

Data display the absolute levels of M1 and all other lysine linkage types. Data are presented as means ± SEM; each $n = 3$ represents an individual biological replicate; P-values were determined by two-tailed Student's t-test; ns depicts not significant ($P > 0.05$); *: $P < 0.05$; **: $P < 0.01$; ***: $P < 0.001$; ****: $P < 0.0001$.

 © 2018 The Authors

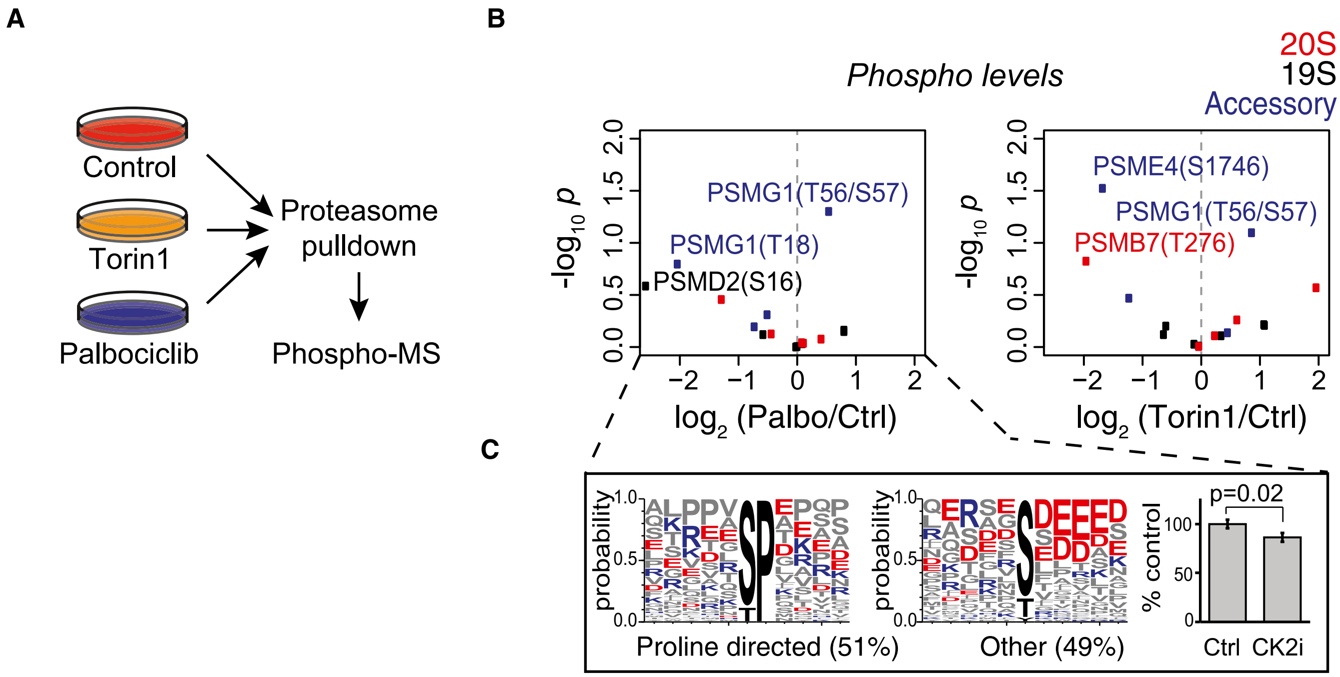

Figure EV6. Quantitative mass spectrometry of proteasome pulldowns.

A Workflow schematic of the mass spectrometry-based analysis of proteasome activation mechanism. Palbociclib (10 μM) and Torin-1 (1 μM) treatments of MCF7 cells lasted 4 h (n = 3).

B Volcano plots showing abundance changes and statistical significances for phosphorylation sites in proteasome subunits. Palbociclib-induced effects are on the left, Torin-1 on the right.

C Motif analysis of palbociclib-induced changes in proteasomal phosphopeptides. Bar chart on right displays proteasome activity levels in MCF7 cells after 2-h incubation with 5 μM casein kinase 2 inhibitor quinalizarin (CK2i) (n = 3).

Data information: In panel (C), data are presented as means ± SD; each n represents an individual biological replicate; P-value was determined by two-tailed Student's t-test.

Figure EV7. ECM 29 and Kaplan–Meier survival curves for additional datasets.

A Proteasome activity levels, as measured by Me4BodipyFL-Ahx3Leu3VS probe, after siRNA-mediated knockdown of ECM29 and subsequent 6-h treatment with palbociclib (1 μM) or Torin-1 (0.1 μM) in HeLa cells (n = 4).

B MCF7 (left) and T47D (right) proliferation after ECM29 or control siRNAs transfection [n = 4 (MCF7) or 3 (T47D)]. Western blots display the knockdown efficiency after 48 h.

C ECM29 expression in GSE37751 dataset (Terunuma *et al*, 2014) (left) and ECM29 expression in TCGA breast cancer data. The survival data are from PROGgeneV2—Pan Cancer Prognostics Database (http://watson.compbio.iupui.edu/chirayu/proggene/database/index.php). Statistical analysis of the Kaplan–Meier (KM) plots has been described (Goswami & Nakshatri, 2014).

Data information: In panels (A and B), data are presented as means ± SD; each n represents an individual biological replicate; P-values were determined by ANOVA and two-tailed Student's t-test with Holm–Sidak *post hoc* test.

© 2018 The Authors

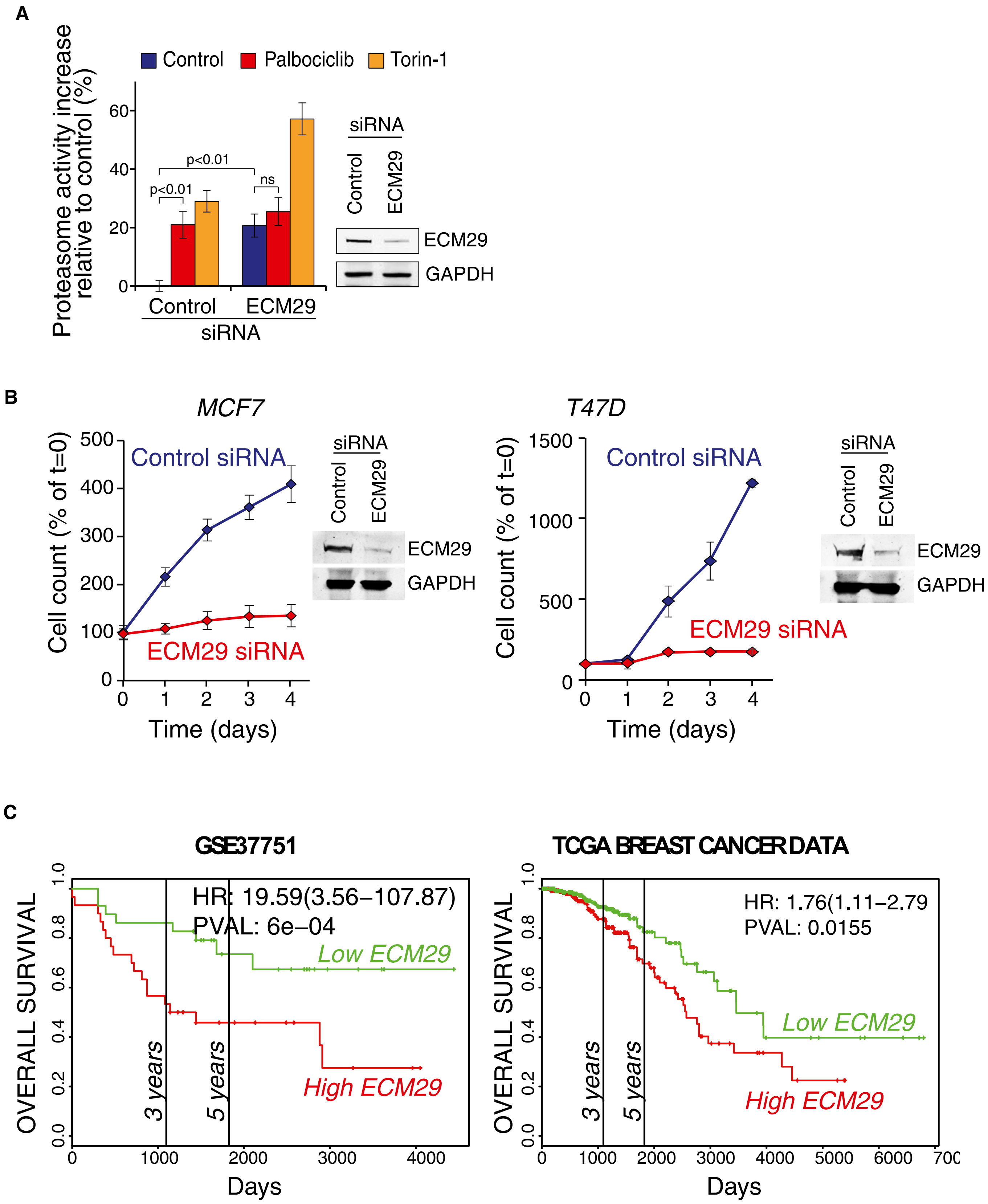

Figure EV7.

© 2018 The Authors
