# Supplementary material for: Thermal proteome profiling of breast cancer cells reveals proteasomal activation by CDK4/6 inhibitor palbociclib
Source: EMBO J. 2018 Apr 18;37(10):e98359. doi: 10.15252/embj.201798359 (PMC5978322; doi:10.15252/embj.201798359)
Supplement: Supplementary file 8 — Source Data for Figure 1 [file EMBJ-37-e98359-s007.pdf]

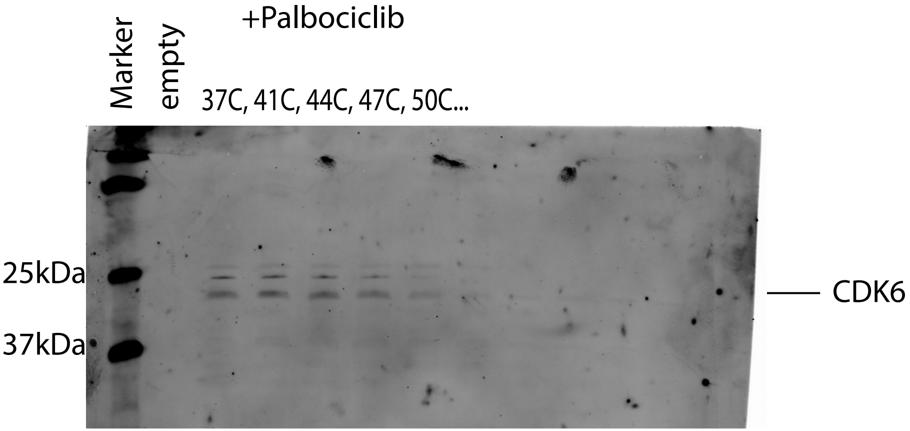

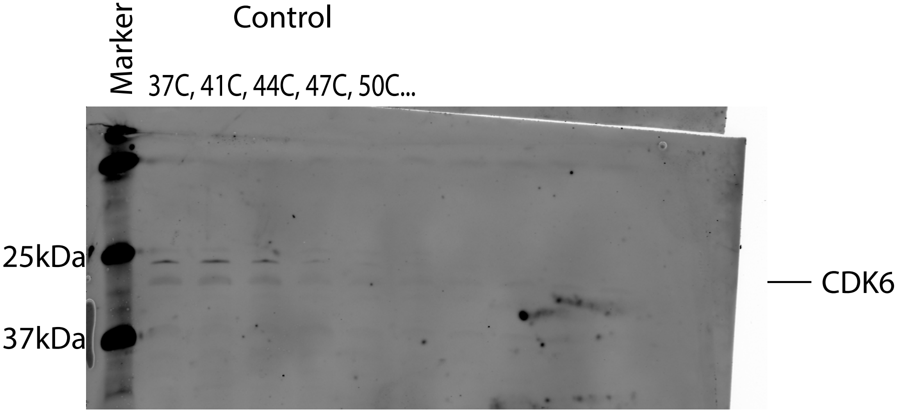

The second band above CDK6 (smaller protein, images unrotated) is most likely CDK4, which was analysed first, before stripping the blot and analysing for CDK6. In all samples, CDK6 WB band intensity was very weak.
