# Supplementary figures and images for: Thermal proteome profiling of breast cancer cells reveals proteasomal activation by CDK4/6 inhibitor palbociclib
Source: EMBO J. 2018 Apr 18;37(10):e98359. doi: 10.15252/embj.201798359 (PMC5978322; doi:10.15252/embj.201798359)

Figure 4b plots

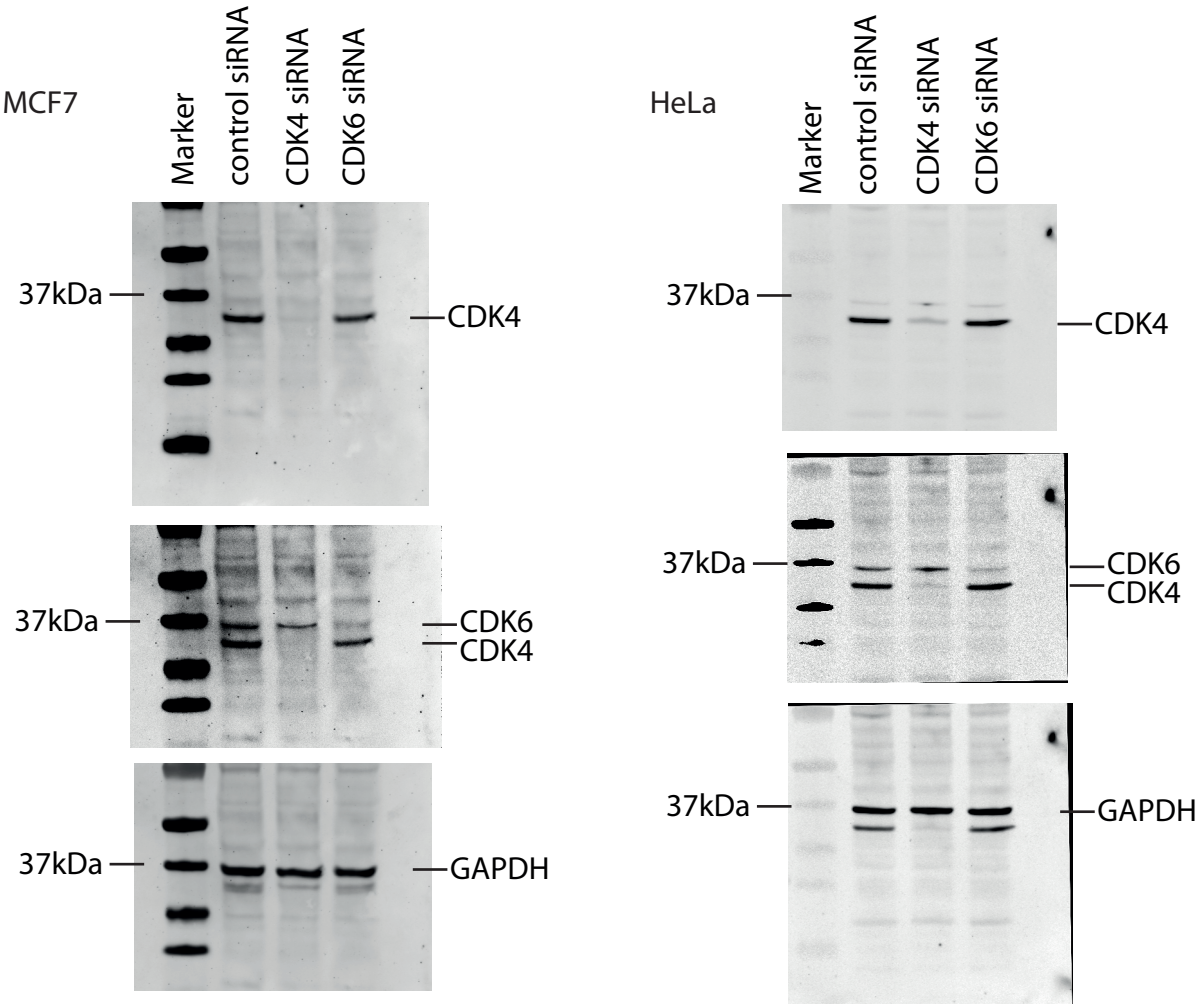

Figure 4c plots

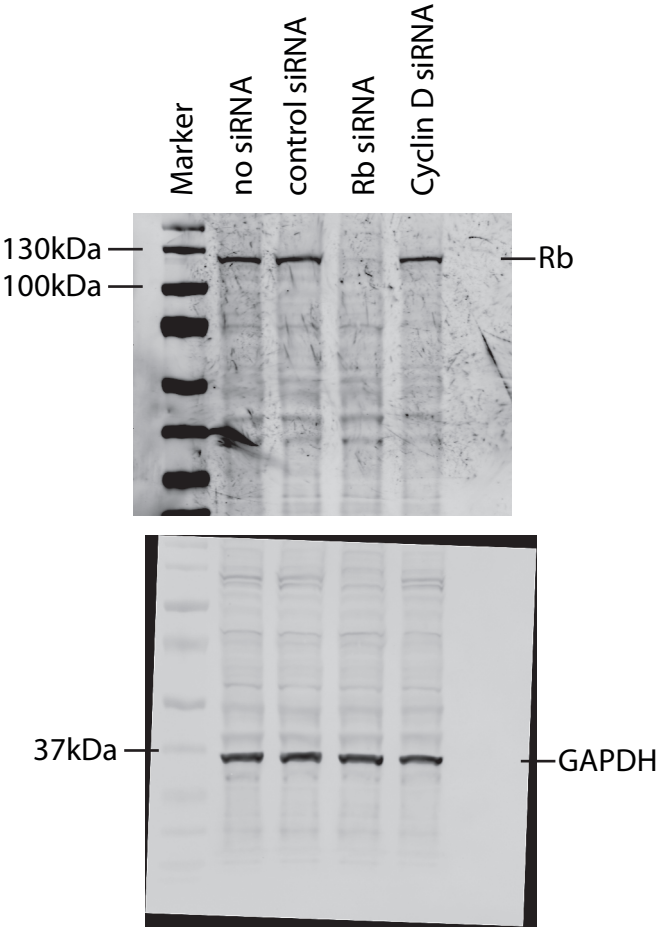

Supplement: Supplementary file 9 — Source Data for Figure 4 [file EMBJ-37-e98359-s008.pdf]

Figure 5a plots

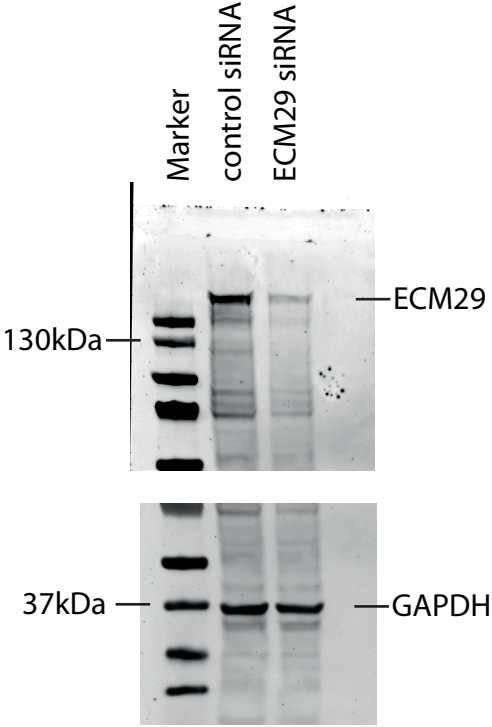

Figure 5b plots

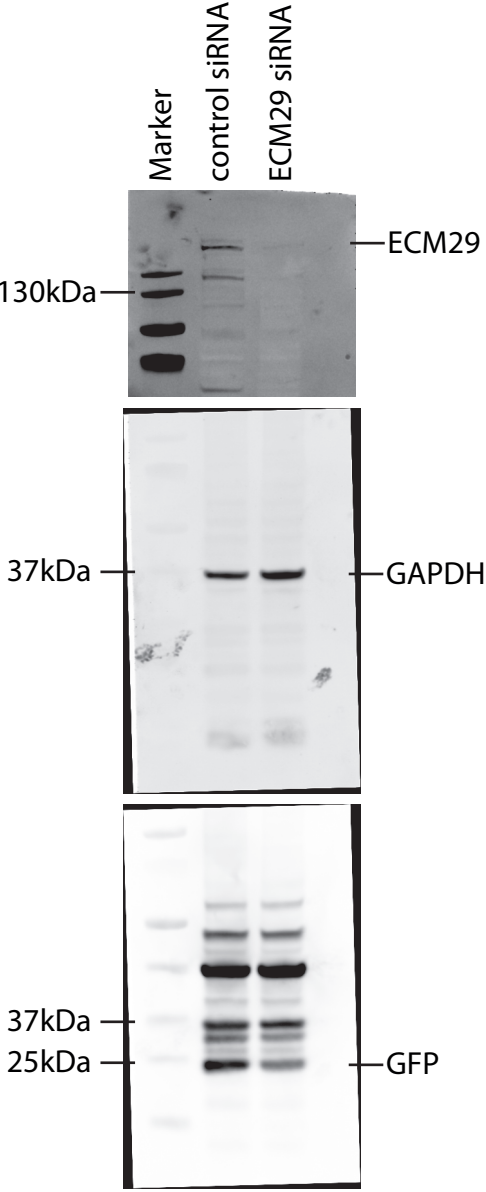

Supplement: Supplementary file 10 — Source Data for Figure 5 [file EMBJ-37-e98359-s009.pdf]

Figure 6a plots

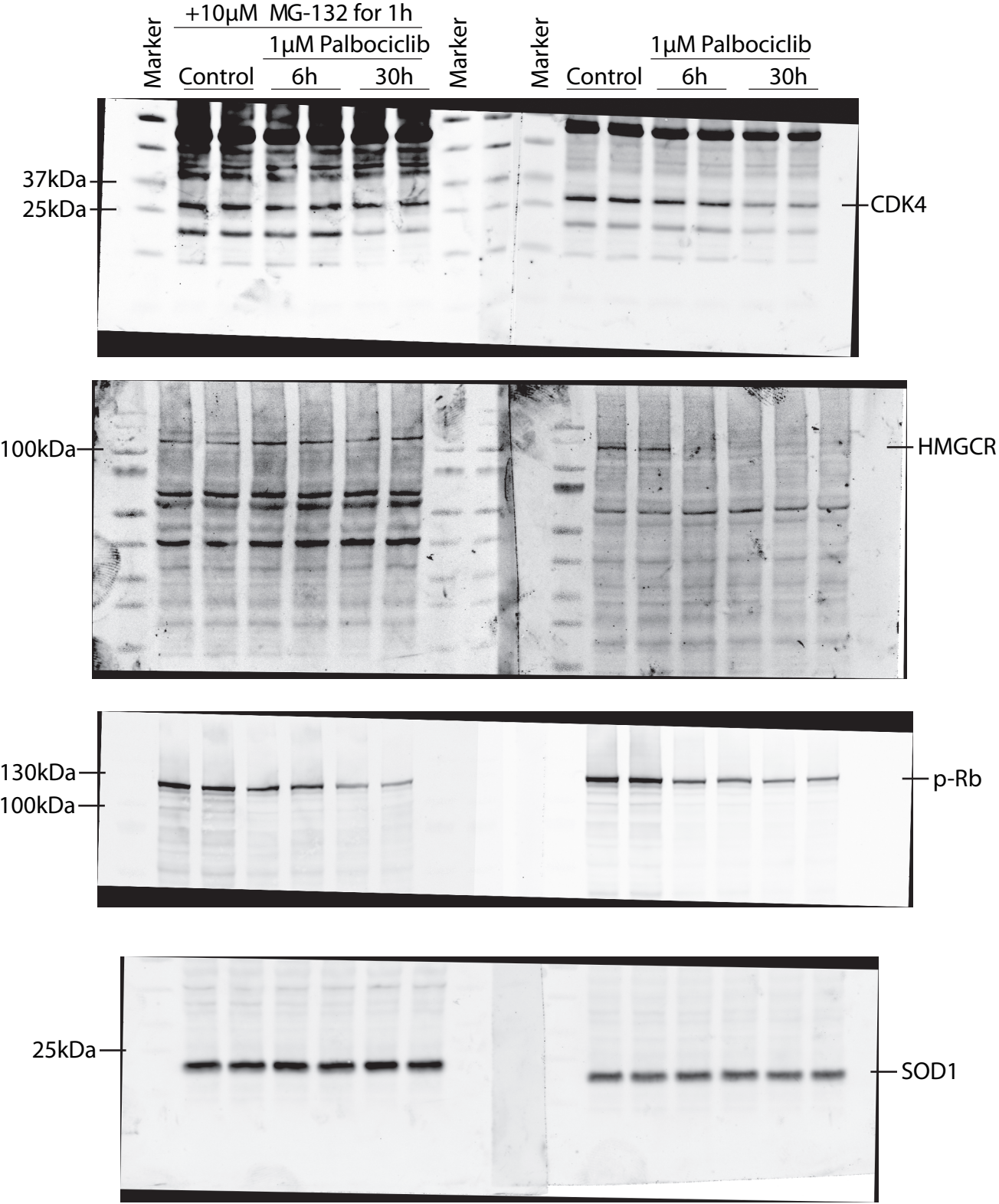

Supplement: Supplementary file 11 — Source Data for Figure 6 [file EMBJ-37-e98359-s010.pdf]
